# Mathematical modelling and sliding characteristics analysis on the spherical movable teeth for the toroidal drive

**DOI:** 10.1038/s41598-025-95352-9

**Published:** 2025-04-01

**Authors:** Xuelian Zeng, Ligang Yao, Meiyan Lou

**Affiliations:** 1 School of Automobile Engineering, Fujian Chuanzheng Communications College, Fuzhou, 350007 China; 2 School of Mechanical Engineering and Automation, Fuzhou University, Fuzhou, 350116 China; 3 Fuzhou Polytechnic, Fuzhou, 350116 China

**Keywords:** Toroidal drive, Rolling–sliding, Sliding velocity, Mathematical modelling, Contact analysis, Engineering, Mechanical engineering

## Abstract

Toroidal drives combine a planetary gear drive with a worm gear drive. Furthermore, in toroidal drives, the planetary gear teeth are movable. The motion of spherical movable teeth for planetary gears affects the friction, wear and transmission efficiency of the meshing surface, but this effect has not been thoroughly studied to date. This study involved a kinematic analysis of planetary gear teeth. Frenet-fram was first introduced into the toroidal drive to describe the motion of spherical movable teeth. A contact analysis of the spherical movable teeth was carried out, and a mathematical model of rolling–sliding motion between the spherical movable teeth and the central worm spiral groove was established via the conversion mechanism method. The instantaneous velocity formula and relative sliding velocity formula at the meshing points of movable teeth were derived. The influence of the system parameters on the sliding velocity was analysed, and several useful conclusions were drawn. These results can provide a theoretical foundation for subsequent research on the friction loss and transmission efficiency of toroidal drives.

## Introduction

A toroidal drive is a type of transmission mechanism that combines the positive attributes of a planetary gear drive and a circular worm drive due to the introduction of movable teeth in meshing contact with rolling movement between the central worm and planetary gears and between the stationary internal gear and planetary gears^1^. Most previous studies on toroidal drives have focused on developing theoretical models for meshing, wear and manufacturing. The meshing process of toroidal drives has been investigated, and varying meshing characteristics across different roller shapes, including conical rollers, cylindrical rollers and spherical rollers, have been analysed^2^. Xu et al.^3,4^ examined the elastic deformation and friction coefficients between the planet gear and the stationary internal gear or the worm and then deduced the contact stress distributions and wear formulas of the toroidal drive. Furthermore, to solve the problem of machining spiral raceways of internal gears, Yao et al.^5,6^ proposed enveloping and NC machining methods.

**Fig. 1:**
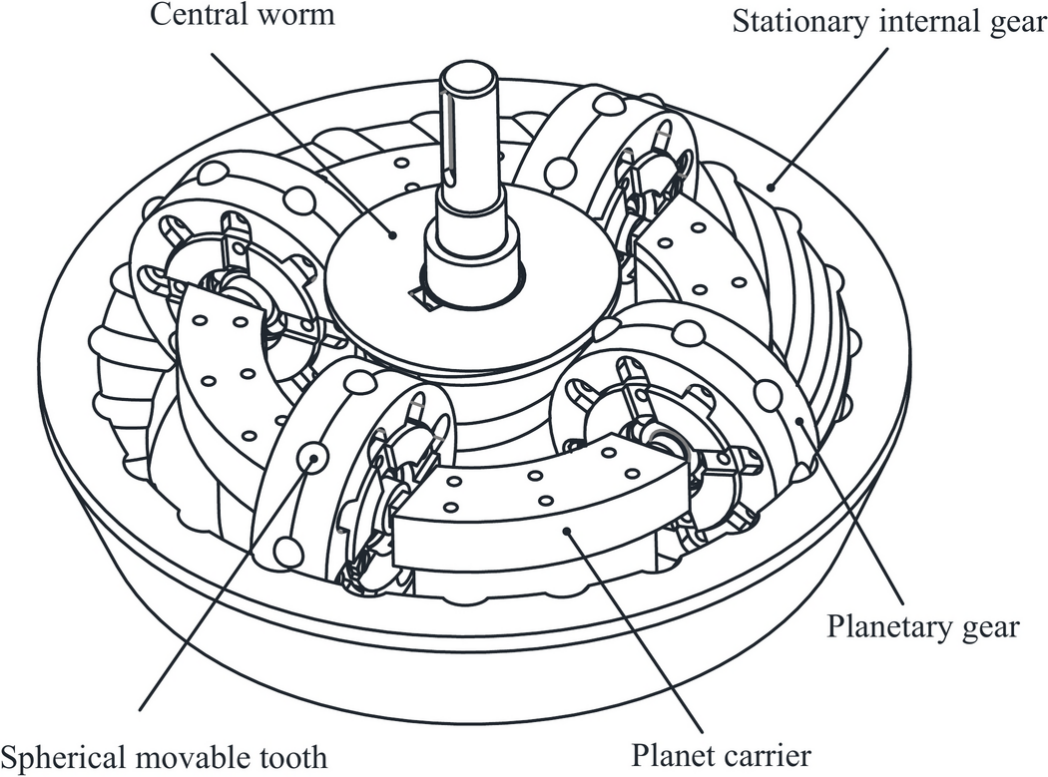
Schematic structure of a toroidal drive with spherical movable teeth.

In a toroidal drive (Fig. 1), the motion of spherical movable teeth is complex since planetary gears rotate about their own axis and that of the central worm; moreover, movable teeth move along the spiral raceway of the central worm and that of the stationary internal gear. However, previous studies have not elucidated the movement of movable teeth, which has a direct effect on the friction and transmission efficiency of toroidal drives. However, it is useful to consider previous studies on the ball screw mechanism since the structure of the central worm raceway is similar to that of the screw, and the motion of the spherical movable teeth is similar to that of the balls, which includes both rolling and sliding.

The spin, rolling and sliding velocities coexist at the ball‒groove interface of the ball-screw mechanism^7^. To study the kinematics of the ball screw mechanism, Lin et al.^8,9^ derived the slip velocities of the ball and developed theories for evaluating the efficiency of the ball screw mechanism by affixing a Frenet-coordinate system to the path of the ball centre. By using a Frenet-coordinate system to describe the path of the ball centre, Wei and Lin^10^ evaluated the influence of different parameters, such as the friction coefficient, normal force acting on the ball, and contact angle, on a ball-screw mechanism at two contact areas. Additionally, by setting up four coordinate systems, Wei and Lai^11^ analysed the kinematics and transmission efficiency of a preloaded single-nut, double-cycle ball screw operating at high rotational speeds, which included a Frenet-coordinate system. Xu and Tang^12^ first established a global coordinate system and Frenet-coordinate system, set up a ball sliding model, and analysed the sliding characteristics caused by different factors for a ball screw operating at high speeds. Xu et al.^13^ developed a new systematic creep analysis model to calculate the friction of a ball screw by establishing a properly transformed coordinate system. Hu et al.^14^ discussed 5 possible relative motion states in a ball screw mechanism and analysed the kinematic characteristics and slip‒roll ratios at the contact points of the raceway by establishing a proper coordinate system, which included a Frenet-coordinate system. Cheng et al.^15^ developed a motion model to describe the dynamic sliding and rolling behaviours between a ball and a raceway. These studies provide a reference for developing a proper coordinate system to establish a kinematic model of movable teeth in toroidal drives. Du et al.^16^ studied the kinematics of a ball screw mechanism to expound the dynamic process of the drive and analysed the influence of contact deformation on the dynamic characteristics. Zhen and An^17^ established a model to calculate the contact stress and fatigue life of ball screws via mechanics analysis and analysed the influences of the axial load, radial load and size accuracy of balls on the fatigue life of ball screws. Brecher et al.^18^ introduced a methodology to calculate the load distribution and rolling element contact characteristics of a ball screw drive and then experimentally measured the load‒displacement characteristics to validate the calculation methodology. Bertolino et al.^19^ developed a high-fidelity dynamic model of the ball screw, which can represent the roll/slip transition in the sphere/groove contact points depending on the dynamic operating conditions. Wang et al.^20^ proposed an approach to obtain the slide–roll ratio and entrainment velocity of ball screws. Then, the effects of structural factors on the entrainment velocity and slide–roll ratio were analysed. Research on the rolling‒sliding motion of ball screws can provide a reference for the kinematic analysis of toroidal drives.

In this work, a mathematical sliding model is developed for spherical movable teeth during conjugate contact with the spiral groove of the central worm in a toroidal drive. The influences of structural parameters on the sliding velocity of movable teeth are analysed and discussed.

## Coordinate system establishment

**Fig. 2:**
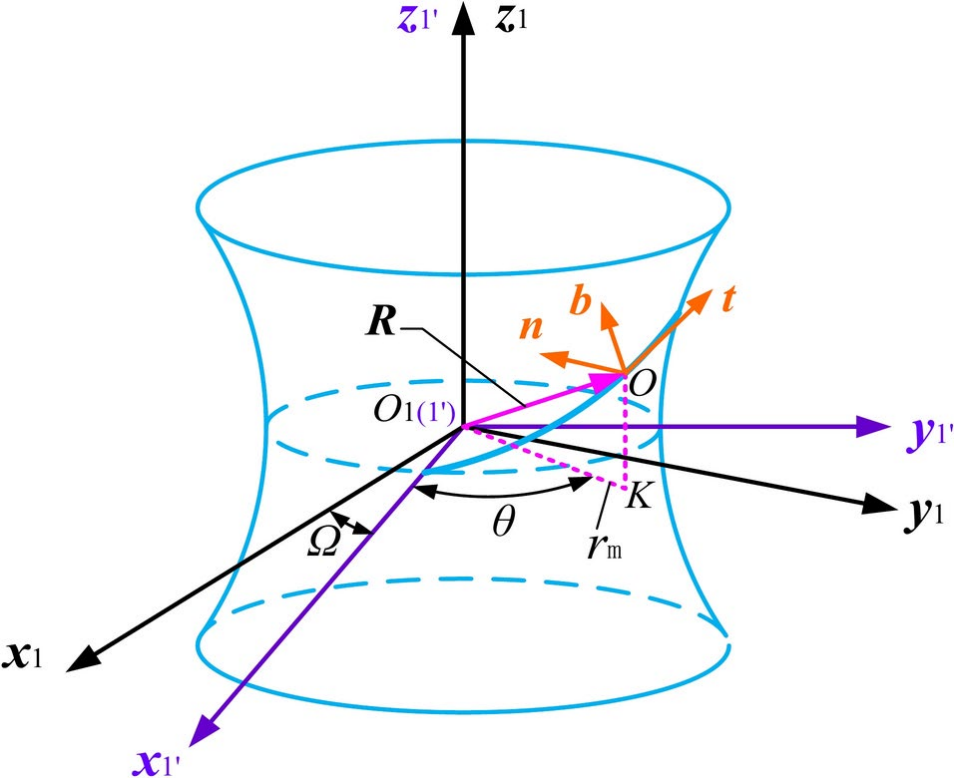
Three sets of coordinate systems: World (black), Rotating (purple) and Frenet (orange).

To study the kinematics of spherical movable teeth, a Frenet-coordinate system is affixed to the path of the movable tooth centre. As shown in Fig. 2, three sets of coordinate systems are introduced. The first coordinate system (world), S_1_(*O*_1_*,x*_1_*,y*_1_*,z*_1_), originates at the centre of the central worm, and the *z*_1_-axis is coincident with the axis of the central worm. The second coordinate system (rotating), S_1′_(*O*_1′_*,x*_1′_*,y*_1′_*,z*_1′_), is fixed with its *z*_1′_-axis coincident with the central worm axis and rotates about the *z*_1′_-axis. The third coordinate system (Frenet), S_0_(*O*,*t*,*n*,*b*), originates at the centre of the movable tooth for the planetary gear and moves with it. The *t*-axis coincides with the tangent direction of the movable tooth moving in the spiral raceway of the central worm. The *n*-axis is perpendicular to the central worm surface and intersects with the *z*_1′_-axis. The *K* point is the projection of the movable tooth centre (*O*) on the *x*_1′_–*y*_1′_ plane, and *θ* is the angle between *O*_1_*K* and the *x*_1′_-axis. We define the plane composed of the *b*-axis and *n*-axis as the normal plane of the movable tooth and that composed of the *t*-axis and* b*-axis as the tangent plane of the movable tooth. The contact point between the movable tooth and the helical tooth of the central worm is always located in the normal plane.

The relationship between the rotating coordinate system S_1′_ and the world coordinate system S_1_ can be determined based on Fig. 2. It can be expressed as:

1 $$S_{1^{\prime}} = {\varvec{M}}_{1\prime 1} \cdot S_{1}$$

where ***M***_1′1_ is the coordinate transformation matrix from S_1_ to S_1′_,

2 $${\varvec{M}}_{1^{\prime}1} = \left[ {\begin{array}{*{20}c} {C_{\mathit{\Omega} } } & {S_{\mathit{\Omega} } } & 0 \\ { - S_{\mathit{\Omega} } } & {C_{\mathit{\Omega} } } & 0 \\ 0 & 0 & 1 \\ \end{array} } \right]$$

where *C*_*Ω*_** = ***cos*(*Ω*), *S*_*Ω*_** = ***sin*(*Ω*) and *Ω* is the angular displacement between S_1_ and S_1′_.

Assume that a movable tooth moves from the *x*_1′_–_1′_ plane along the helical groove of the central worm by a *θ* angle. If the lead of the central worm is *L*, then the position vector ^*r*^***R*** of the movable tooth centre with respect to S_1′_ can be expressed as:

3 $${}^{r}{\varvec{R}} = {\varvec{R}}^{T} S_{1^{\prime}}$$

4 $${\varvec{R}}^{T} = [\begin{array}{*{20}c} {r_{m} C_{\theta } } & {r_{m} S_{\theta } } & {r_{m} \theta t_{\lambda } } \\ \end{array} ]$$

where *r*_*m*_ is the instantaneous radius of the spherical movable tooth trajectory, *λ* is the corresponding spiral angle of the central worm, and *t*_*λ*_ = *tan*(*λ*) = *L*/2π*r*_*m****.***_

Combining Eqs. (1) and (3) with Eq. (4), the position vector of point *O* relative to S_1_ can be obtained:

5 $${}^{w}{\varvec{R}} = {\varvec{R}}^{T} {\varvec{M}}_{1\prime 1} S_{1} .$$

The trajectory curve of the movable tooth centre can be expressed as:

6 $${\varvec{r}}(t) = \left[ {\begin{array}{*{20}c} {r_{m} C_{\theta } } & {r_{m} S_{\theta } } & {r_{m} \theta t_{\lambda } } \\ \end{array} } \right]S_{1^{\prime}} .$$

In the Frenet coordinate system S_0_,

7 $$\left\{ {\begin{array}{*{20}l} {\user2{t}=\frac{{{\varvec{r}}^{\prime}}}{{\left| {{\varvec{r}}^{\prime}} \right|}}} \hfill \\ {\user2{n}=\frac{{{\varvec{r}}^{\prime} \times {\varvec{r}}^{\prime\prime}}}{{\left| {{\varvec{r}}^{\prime} \times {\varvec{r}}^{\prime\prime}} \right|}}} \hfill \\ {\user2{b = t} \times {\varvec{n}}} \hfill \\ \end{array} } \right.$$

that is,

8 $$\left\{ {\begin{array}{*{20}l} {{\varvec{t}} = \left[ {\begin{array}{*{20}c} { - S_{\theta } C_{\lambda } } & {C_{\theta } C_{\lambda } } & {S_{\lambda } } \\ \end{array} } \right]S_{1^{\prime}} } \hfill \\ {{\varvec{n}} = \left[ {\begin{array}{*{20}c} {S_{\theta } S_{\lambda } } & { - C_{\theta } S_{\lambda } } & {C_{\lambda } } \\ \end{array} } \right]S_{1^{\prime}} } \hfill \\ {{\varvec{b}} = \left[ {\begin{array}{*{20}c} {C_{\theta } } & {S_{\theta } } & 0 \\ \end{array} } \right]S_{1^{\prime}} } \hfill \\ \end{array} } \right.$$

According to differential geometry, the trajectory curve of the movable tooth centre can be written as:

9 $$d= \left| {{\varvec{r}}^{\prime}} \right| = \frac{{r_{m} }}{{C_{\lambda } }}$$

the curvature and torsion can be expressed as:

10 $$k = \frac{{\left| {{\varvec{r}}^{\prime} \times {\varvec{r}}^{\prime\prime}} \right|}}{{\left| {{\varvec{r}}^{\prime}} \right|^{3} }} = \frac{{C_{\lambda }^{2} }}{{r_{m} }}$$

11 $$\tau = \frac{{\left| {(\user2{r}',\user2{r}'',\user2{r}''')} \right|}}{{\left| {\user2{r}' \times \user2{r}''} \right|^{2} }} = \frac{{S_{\lambda } C_{\lambda } }}{{r_{m} }}$$

The curvature and torsion are only related to *r*_*m*_ and *λ*, not to *θ*.

Then, Eq. (8) can be rewritten as:

12 $$\left\{ {\begin{array}{*{20}l} {{\varvec{t}} = \left[ {\begin{array}{*{20}c} { - kdS_{\theta } } & {kdC_{\theta } } & {\tau d} \\ \end{array} } \right]S_{1^{\prime}} } \hfill \\ {{\varvec{n}} = \left[ {\begin{array}{*{20}c} {\tau dS_{\theta } } & { - \tau dC_{\theta } } & {kd} \\ \end{array} } \right]S_{1^{\prime}} } \hfill \\ {{\varvec{b}} = \left[ {\begin{array}{*{20}c} {C_{\theta } } & {S_{\theta } } & 0 \\ \end{array} } \right]S_{1^{\prime}} } \hfill \\ \end{array} } \right.$$

The relationship between the Frenet coordinate system S_0_ and the rotating coordinate system S_1′_ can be determined based on Fig. 2, as follows:

13 $$S_{0} = {\varvec{M}}_{01^{\prime}} \cdot S_{1^{\prime}}$$

14 $${\varvec{M}}_{01^{\prime}} = \left[ {\begin{array}{*{20}c} { - S_{\theta } C_{\lambda } } & {C_{\theta } C_{\lambda } } & {S_{\lambda } } \\ {S_{\theta } S_{\lambda } } & { - C_{\theta } S_{\lambda } } & {C_{\lambda } } \\ {C_{\theta } } & {S_{\theta } } & 0 \\ \end{array} } \right].$$

Then, Eq. (3) can be expressed as:

15 $${}^{r}{\varvec{R}} = {\varvec{R}}^{T} {\varvec{M}}_{1^{\prime}0} S_{0} = r_{m} \left[ {\begin{array}{*{20}c} {\theta t_{\lambda } S_{\lambda } } & {\theta S_{\lambda } } & 1 \\ \end{array} } \right]S_{0} .$$

The relationship between the Frenet coordinate system S_0_ and the world coordinate system S_1_ can be written as:

16 $$S_{0} = \left[ {\begin{array}{*{20}c} { - S_{{\theta { + }\mathit{\Omega} }} C_{\lambda } } & {C_{{\theta { + }\mathit{\Omega} }} C_{\lambda } } & {S_{\lambda } } \\ {S_{{\theta { + }\mathit{\Omega} }} S_{\lambda } } & { - C_{{\theta { + }\mathit{\Omega} }} S_{\lambda } } & {C_{\lambda } } \\ {C_{{\theta { + }\mathit{\Omega} }} } & {S_{{\theta { + }\mathit{\Omega} }} } & 0 \\ \end{array} } \right]S_{1} .$$

The variation rate of the Frenet frame can be obtained via the derivative of Eq. (16) with respect to time, as follows:

17 $$S^{\prime}_{0} = \left( {\theta ^{\prime} + \mathit{\Omega} ^{\prime}} \right)\left[ {\begin{array}{*{20}c} { - C_{{\theta { + }\mathit{\Omega} }} C_{\lambda } } & { - S_{{\theta { + }\mathit{\Omega} }} C_{\lambda } } & 0 \\ {C_{{\theta { + }\mathit{\Omega} }} S_{\lambda } } & {S_{{\theta { + }\mathit{\Omega} }} S_{\lambda } } & 0 \\ { - S_{{\theta { + }\mathit{\Omega} }} } & {C_{{\theta { + }\mathit{\Omega} }} } & 0 \\ \end{array} } \right]S_{1} .$$

**Fig. 3:**
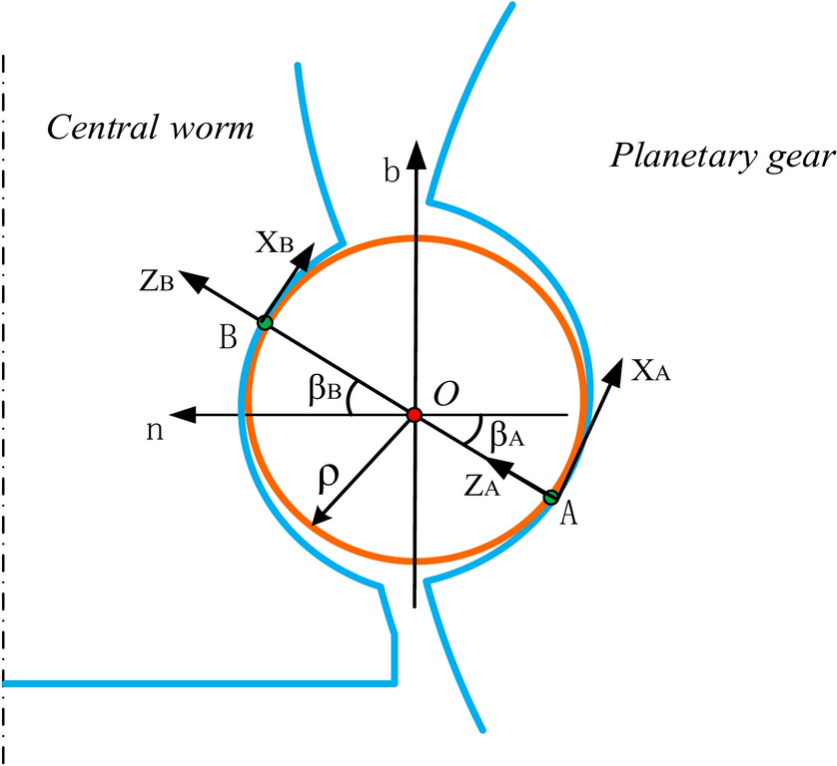
Positions of the contact points in the normal plane.

The movable tooth can only move in the tangent direction of the Frenet coordinate system **S**_0_ with respect to the central worm, meaning that the contact point must be on the normal plane. To locate contact points, the concept of the contact angle is introduced. As shown in Fig. 3, the contact angle *β* is defined as the angle between the unit normal vector and the contact vector. Notably, *β* is considered positive when it is measured clockwise from the positive side of the normal axis.

Without loss of generality, assume that points *A* and *B* are the instantaneous contact points between the movable tooth and the planetary gear and central worm, respectively. To describe the position of the contact points, additional coordinate systems (*i*, X_*i*_, Y_*i*_, Z_*i*_), *i* = A, B, are introduced. The X_*i*_–Y_*i*_ plane lies on the contact plane, and the Z_*i*_ axis coincides with the common normal direction of the two contacting bodies.

The relationship between the coordinate system S_i_ and the Frenet coordinate system S_0_ can be written as:

18 $$S_{i} = {\varvec{M}}_{i0} \cdot S_{0}$$

where

19 $$S_{i} = \left[ {\begin{array}{*{20}c} {{\varvec{i}}_{i} } & {{\varvec{j}}_{i} } & {{\varvec{k}}_{i} } \\ \end{array} } \right]^{T} ,{\varvec{M}}_{i0} = \left[ {\begin{array}{*{20}c} 0 & { - S_{\beta i} } & {C_{\beta i} } \\ 1 & 0 & 0 \\ 0 & {C_{\beta i} } & {S_{\beta i} } \\ \end{array} } \right].$$

Therefore, the position vectors of points A and *B* relative to the movable tooth centre can be described as:

20 $$\left\{ {\begin{array}{*{20}c} {\user2{R}_{{\text{AO}}} = \left[ {\begin{array}{*{20}c} 0 & 0 & \rho \\ \end{array} } \right]S_{\text{A}} } \\ {\user2{R}_{{\text{BO}}} = \left[ {\begin{array}{*{20}c} 0 & 0 & \rho \\ \end{array} } \right]S_{\text{B}} } \\ \end{array} } \right.$$

where *ρ* is the radius of the movable tooth.

The position vectors of points A and *B* relative to the rotating coordinate system can be expressed as:

21 $$\left\{ {\begin{array}{*{20}c} {{}^{r}{\varvec{R}}_{\text{A}} = {}^{r}{\varvec{R}} + {\varvec{R}}_{{\text{AO}}} } \\ {{}^{r}{\varvec{R}}_{\text{B}} = {}^{r}{\varvec{R}} + {\varvec{R}}_{{\text{BO}}} } \\ \end{array} } \right..$$

## Rolling‒sliding mathematical model of movable teeth

The motion of movable teeth along a spiral raceway is complicated and does not only involve pure rolling. To study the kinematics of movable teeth in the conjugate process among the teeth of the toroidal drive, the sliding conditions between the movable teeth and the central worm should first be determined. To determine the sliding velocity of movable teeth, a complete velocity analysis of spherical movable teeth, planetary gears and central worms must be carried out.

The velocity of the movable tooth with respect to the rotating coordinate system S_1′_ can be acquired by differentiating Eq. (15) with respect to time:

22 $${}^{r}{\varvec{R}}^{\prime} = r_{m} \theta ^{\prime}\left[ {\begin{array}{*{20}c} {t_{\lambda } S_{\lambda } } & {S_{\lambda } } & 0 \\ \end{array} } \right]S_{0}$$

Assuming that Ω^′^ ***k*** is the angular velocity of the central worm, the velocity of the movable tooth centre relative to the world coordinate system S_1_ can be described as:

23 $${}^{w}{\varvec{R}}^{\prime} = {}^{r}{\varvec{R}}^{\prime} + \mathit{\Omega} ^{\prime} \times {}^{r}{\varvec{R}} = r_{m} \left[ {\begin{array}{*{20}c} {t_{\lambda } S_{\lambda } \theta ^{\prime} + C_{\lambda } \mathit{\Omega} ^{\prime}} \\ {S_{\lambda } \theta ^{\prime} - S_{\lambda } \mathit{\Omega} ^{\prime}} \\ 0 \\ \end{array} } \right]S_{0}$$

where

24 $$\left\{ {\begin{array}{*{20}l} {\mathit{\Omega} ^{\prime} = \left[ {\begin{array}{*{20}c} {\mathit{\Omega} ^{\prime}S_{\lambda } } & {\mathit{\Omega} ^{\prime}C_{\lambda } } & 0 \\ \end{array} } \right]S_{0} } \hfill \\ {\mathit{\Omega} ^{\prime} \times {}^{r}R = \left[ {\begin{array}{*{20}c} {r_{m} C_{\lambda } \mathit{\Omega} ^{\prime}} & { - r_{m} S_{\lambda } \mathit{\Omega} ^{\prime}} & 0 \\ \end{array} } \right]S_{0} } \hfill \\ \end{array} } \right.$$

Assume that ***ω* = **[*ω*_*t*_* ω*_*n*_* ω*_*b*_]S_0_ is the angular velocity of the movable tooth rotating about the *t*-axis, *n*-axis and *b*-axis, respectively, in the Frenet coordinate system S_0_; then, the instantaneous velocity of the movable tooth at contact points A and B can be described as:

25 $${\varvec{V}}_{{\text{Ar}}} = {}^{w}{\varvec{R}}^{\prime}+\user2{\omega } \times {\varvec{R}}_{{\text{AO}}} = \left[ {\begin{array}{*{20}c} {r_{m} (t_{\lambda } S_{\lambda } \theta ^{\prime} + C_{\lambda } \mathit{\Omega} ^{\prime}) + \rho (\omega_{n} S_{{\beta \text{A}}} - \omega_{b} C_{{\beta \text{A}}} )} \\ {r_{m} S_{\lambda } (\theta ^{\prime} - \mathit{\Omega} ^{\prime}) - \rho \omega_{t} S_{{\beta \text{A}}} } \\ {\rho \omega_{t} C_{{\beta \text{A}}} } \\ \end{array} } \right]^{T} S_{0}$$

26 $${\varvec{V}}_{{\text{Br}}} = {}^{w}{\varvec{R}}^{\prime}+\user2{ \omega } \times {\varvec{R}}_{{\text{BO}}} = \left[ {\begin{array}{*{20}c} {r_{m} (t_{\lambda } S_{\lambda } \theta ^{\prime} + C_{\lambda } \mathit{\Omega} ^{\prime}) + \rho (\omega_{n} S_{{\beta \text{B}}} - \omega_{b} C_{{\beta \text{B}}} )} \\ {r_{m} S_{\lambda } (\theta ^{\prime} - \mathit{\Omega} ^{\prime}) - \rho \omega_{t} S_{{\beta \text{B}}} } \\ {\rho \omega_{t} C_{{\beta \text{B}}} } \\ \end{array} } \right]^{T} S_{0}$$

**Fig. 4:**
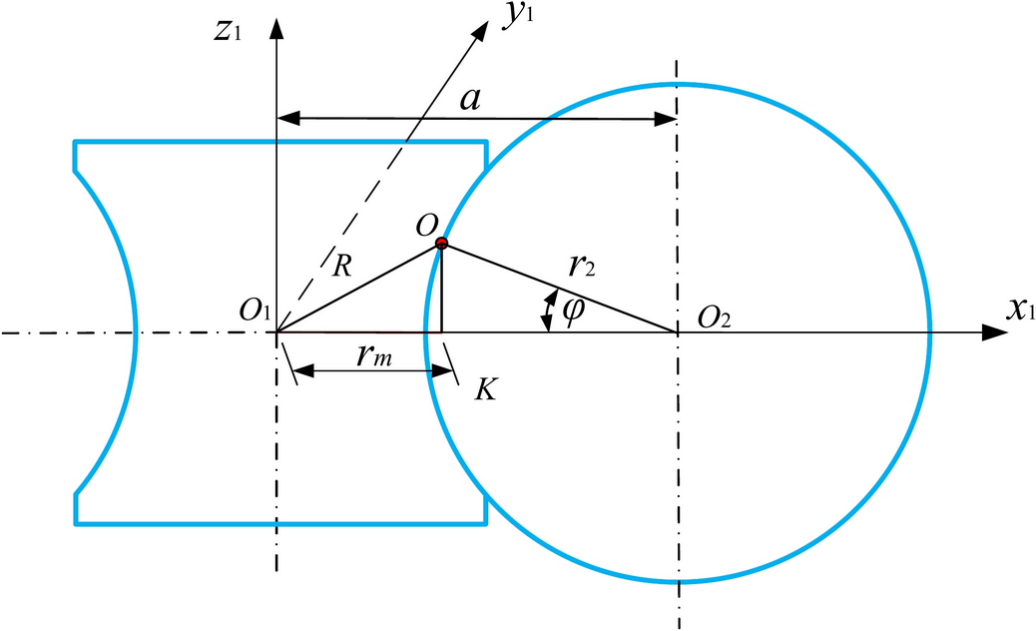
The relationship between the central worm and the planetary gear.

When the central worm rotates in the *Ω* angle, the movable tooth moves to point O along the spiral groove of the central worm from the central section; at the same time, the planetary gear rotates in the *φ* angle, as shown in Fig. 4. Thus,

27 $$r_{m} = a - r_{2} \cdot \cos \varphi$$

28 $$\varphi = \mathit{\Omega} /Z_{2}$$

where *a* is the centre distance between the central worm and the planetary gear, *r*_2_ is the radius of the circle in which the planetary gear teeth centre is located, and Z_2_ is the number of teeth of the planetary gear.

Then, the velocity of the planetary gear at contact point A can be written as:

29 $${\varvec{V}}_{{\text{AW}}} = \mathit{\Omega} {^{\prime}}\left[ {\begin{array}{*{20}c} {\frac{{r_{2} }}{{Z_{2} }} \cdot S_{{\frac{\mathit{\Omega} }{{Z_{2} }}}} } & 0 & {\frac{{r_{{2}} }}{{Z_{{2}} }} \cdot C_{{\frac{\mathit{\Omega} }{{Z_{{2}} }}}} } \\ \end{array} } \right]\text{S}_{{1}} = \mathit{\Omega} {^{\prime}}\left[ {\begin{array}{*{20}c} { - \frac{{r_{{2}} }}{{Z_{{2}} }} \cdot \text{S}_{{\frac{\mathit{\Omega} }{{Z_{{2}} }}}} \text{C}_{\lambda } \text{S}_{{\theta { + }\mathit{\Omega} }} { + }\frac{{r_{{2}} }}{{Z_{{2}} }} \cdot \text{C}_{{\frac{\mathit{\Omega} }{{Z_{{2}} }}}} \text{S}_{\lambda } } \\ {\frac{{r_{{2}} }}{{Z_{{2}} }} \cdot \text{S}_{{\frac{\mathit{\Omega} }{{Z_{{2}} }}}} \text{S}_{\lambda } \text{S}_{{\theta { + }\mathit{\Omega} }} { + }\frac{{r_{{2}} }}{{Z_{{2}} }} \cdot \text{C}_{{\frac{\mathit{\Omega} }{{Z_{{2}} }}}} \text{C}_{\lambda } } \\ {\frac{{r_{{2}} }}{{Z_{{2}} }} \cdot \text{S}_{{\frac{\mathit{\Omega} }{{Z_{{2}} }}}} \text{C}_{{\theta { + }\mathit{\Omega} }} } \\ \end{array} } \right]\text{S}_{{0}}$$

The velocity of the central worm at contact point B can be expressed as:

30 $${\varvec{V}}_{{\text{BW}}} = \mathit{\Omega} {^{\prime}}{\varvec{k}} \times {}^{r}{\varvec{R}}_{\text{B}} = \mathit{\Omega} {^{\prime}}\left[ {\begin{array}{*{20}c} {\text{C}_{\lambda } {(}r_{m} { + }\rho \text{S}_{{\beta \text{B}}} {)}} \\ -{\text{S}_{\lambda } {(}r_{m} { + }\rho \text{S}_{{\beta \text{B}}} {)}} \\ {\rho \text{S}_{\lambda } \text{C}_{{\beta \text{B}}} } \\ \end{array} } \right]\text{S}_{{0}} .$$

The sliding velocity of the movable tooth at contact point A can be described as:

31 $$\begin{aligned} {\varvec{V}}_{{\text{SA}}} & = {\varvec{V}}_{{\text{Ar}}} - {\varvec{V}}_{{\text{Aw}}} \\ & = \left[ {\begin{array}{*{20}c} {r_{m} (t_{\lambda } S_{\lambda } \theta ^{\prime} + C_{\lambda } \mathit{\Omega} ^{\prime}) + \rho (\omega_{n} S_{{\beta \text{A}}} - \omega_{b} C_{{\beta \text{A}}} ) - \mathit{\Omega} ^{\prime}( - \frac{{r_{2} }}{{Z_{2} }}S_{{\frac{\mathit{\Omega} }{{Z_{2} }}}} C_{\lambda } S_{\theta + \mathit{\Omega} } + \frac{{r_{2} }}{{Z_{2} }}C_{{\frac{\mathit{\Omega} }{{Z_{2} }}}} S_{\lambda } )} \\ {r_{m} S_{\lambda } (\theta ^{\prime} - \mathit{\Omega} ^{\prime}) - \rho \omega_{t} S_{{\beta \text{A}}} - \mathit{\Omega} ^{\prime}(\frac{{r_{2} }}{{Z_{2} }}S_{{\frac{\mathit{\Omega} }{{Z_{2} }}}} S_{\lambda } S_{\theta + \mathit{\Omega} } + \frac{{r_{2} }}{{Z_{2} }}C_{{\frac{\mathit{\Omega} }{{Z_{2} }}}} C_{\lambda } )} \\ {\rho \omega_{t} C_{{\beta \text{A}}} - \mathit{\Omega} ^{\prime}\frac{{r_{2} }}{{Z_{2} }}S_{{\frac{\mathit{\Omega} }{{Z_{2} }}}} C_{\theta + \mathit{\Omega} } } \\ \end{array} } \right]^{T} S_{0} \\ & = \left[ {\begin{array}{*{20}c} { - r_{m} S_{\lambda } S_{{\beta \text{A}}} (\theta ^{\prime} - \mathit{\Omega} ^{\prime}) + \rho \omega_{t} + \mathit{\Omega} ^{\prime}\frac{{r_{2} }}{{Z_{2} }}S_{{\beta \text{A}}} (S_{{\frac{\mathit{\Omega} }{{Z_{2} }}}} S_{\lambda } S_{\theta + \mathit{\Omega} } + C_{{\frac{\mathit{\Omega} }{{Z_{2} }}}} C_{\lambda } ) - \mathit{\Omega} ^{\prime}\frac{{r_{2} }}{{Z_{2} }}C_{{\beta \text{A}}} S_{{\frac{\mathit{\Omega} }{{Z_{2} }}}} C_{\theta + \mathit{\Omega} } } \\ {r_{m} (t_{\lambda } S_{\lambda } \theta ^{\prime} + C_{\lambda } \mathit{\Omega} ^{\prime}) + \rho (\omega_{n} S_{{\beta \text{A}}} - \omega_{b} C_{{\beta \text{A}}} ) - \mathit{\Omega} ^{\prime}\frac{{r_{2} }}{{Z_{2} }}(C_{{\frac{\mathit{\Omega} }{{Z_{2} }}}} S_{\lambda } - S_{{\frac{\mathit{\Omega} }{{Z_{2} }}}} C_{\lambda } S_{\theta + \mathit{\Omega} } )} \\ {r_{m} S_{\lambda } C_{{\beta \text{A}}} (\theta ^{\prime} - \mathit{\Omega} ^{\prime}) - \mathit{\Omega} ^{\prime}\frac{{r_{2} }}{{Z_{2} }}C_{{\beta \text{A}}} (S_{{\frac{\mathit{\Omega} }{{Z_{2} }}}} S_{\lambda } S_{\theta + \mathit{\Omega} } + C_{{\frac{\mathit{\Omega} }{{Z_{2} }}}} C_{\lambda } ) - \mathit{\Omega} ^{\prime}\frac{{r_{2} }}{{Z_{2} }}S_{{\beta \text{A}}} S_{{\frac{\mathit{\Omega} }{{Z_{2} }}}} C_{\theta + \mathit{\Omega} } } \\ \end{array} } \right]^{T} S_{\text{A}} \\ & = V_{{\text{SA}}} {\varvec{n}}_{{\text{SA}}} . \\ \end{aligned}$$

**Fig. 5:**
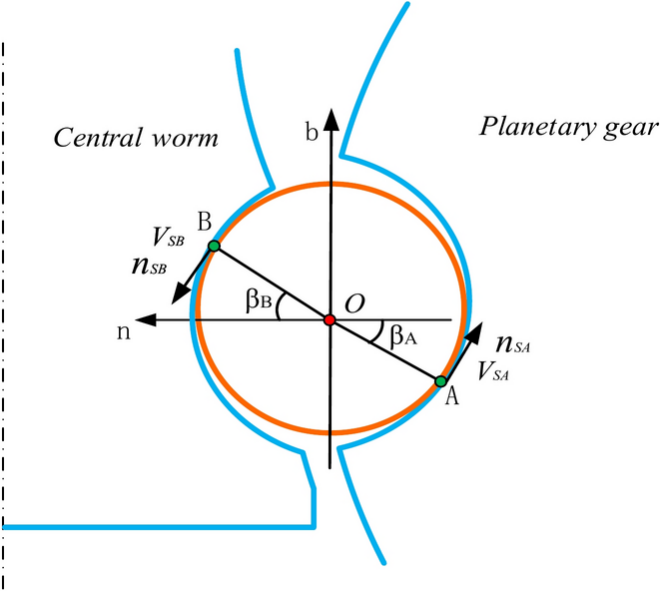
Sliding velocities of contact points A and B*.*

The sliding velocity of the movable tooth at contact point B can be expressed as:

32 $$\begin{aligned} {\varvec{V}}_{{\text{SB}}} & = {\varvec{V}}_{{\text{Br}}} - {\varvec{V}}_{{\text{Bw}}} \\ & = \left[ {\begin{array}{*{20}c} {r_{m} t_{\lambda } S_{\lambda } \theta ^{\prime} + \rho (\omega_{n} S_{{\beta \text{B}}} - \omega_{b} C_{{\beta \text{B}}} ) - \rho \mathit{\Omega} ^{\prime}C_{\lambda } S_{{\beta \text{B}}} } \\ {r_{m} S_{\lambda } \theta ^{\prime} + \rho S_{{\beta \text{B}}} (\mathit{\Omega} ^{\prime}S_{\lambda } - \omega_{t} )} \\ {\rho C_{{\beta \text{B}}} (\omega_{t} - \mathit{\Omega} ^{\prime}S_{\lambda } )} \\ \end{array} } \right]^{T} S_{0} \\ & = \left[ {\begin{array}{*{20}c} { - r_{m} S_{\lambda } \theta ^{\prime}S_{{\beta \text{B}}} - \rho S_{\lambda } \mathit{\Omega} ^{\prime} + \rho \omega_{t} } \\ {r_{m} t_{\lambda } S_{\lambda } \theta ^{\prime} + \rho (\omega_{n} S_{{\beta \text{B}}} - \omega_{b} C_{{\beta \text{B}}} ) - \rho \mathit{\Omega} ^{\prime}C_{\lambda } S_{{\beta \text{B}}} )} \\ {r_{m} S_{\lambda } \theta ^{\prime}C_{{\beta \text{B}}} } \\ \end{array} } \right]^{T} S_{\text{B}} \\ & = V_{{\text{SB}}} {\varvec{n}}_{{\text{SB}}} \\ \end{aligned}$$

where* V*_SA_ and *V*_SB_ are the magnitudes of the sliding velocity, and ***n***_SA_ and ***n***_SB_ are the unit vectors in the direction of the sliding velocities at points A and B, respectively, as shown in Fig. 5. Notably, the unit vectors ***n***_SA_ and ***n***_SB_ are opposite to the direction of the friction force on the movable tooth at contact points A and B, respectively.

## Sliding characteristics analysis

**Fig. 6:**
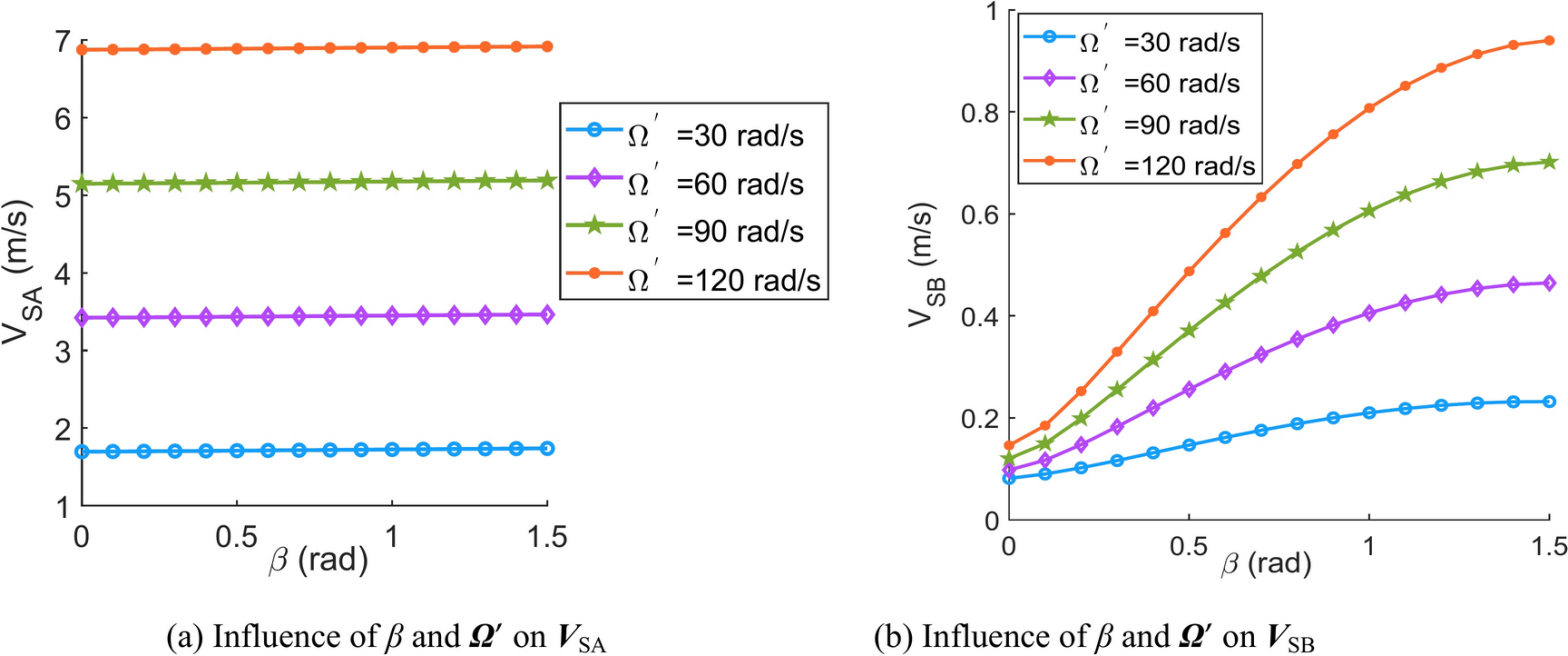
Influence of the contact angle (*β*) and rotation velocity of the central worm (***Ω***^′^) on the sliding velocity of the movable tooth (***V***_S_).

**Fig. 7:**
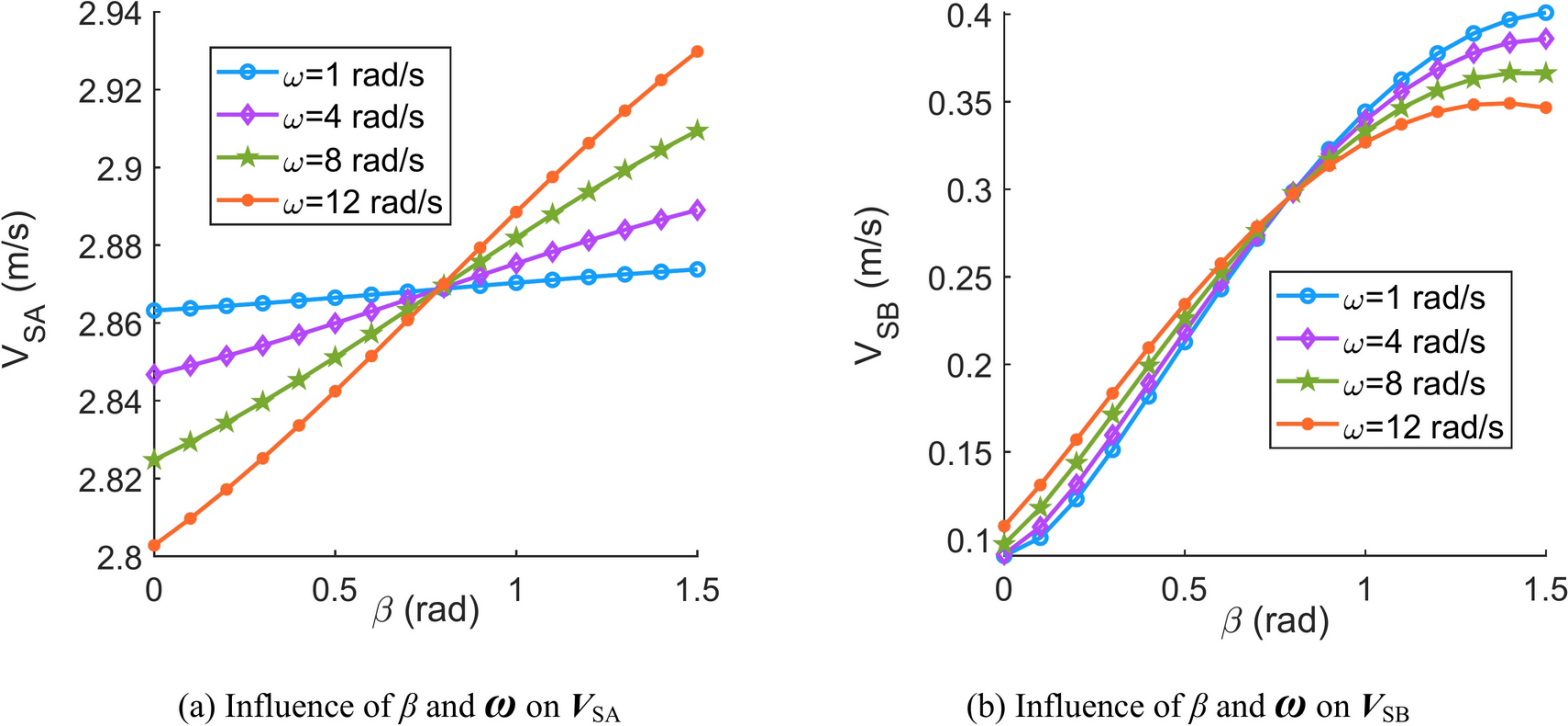
Influence of contact angle (*β*) and spin velocity of the movable tooth (***ω***) on the sliding velocity of the movable tooth (***V***_S_).

**Fig. 8:**
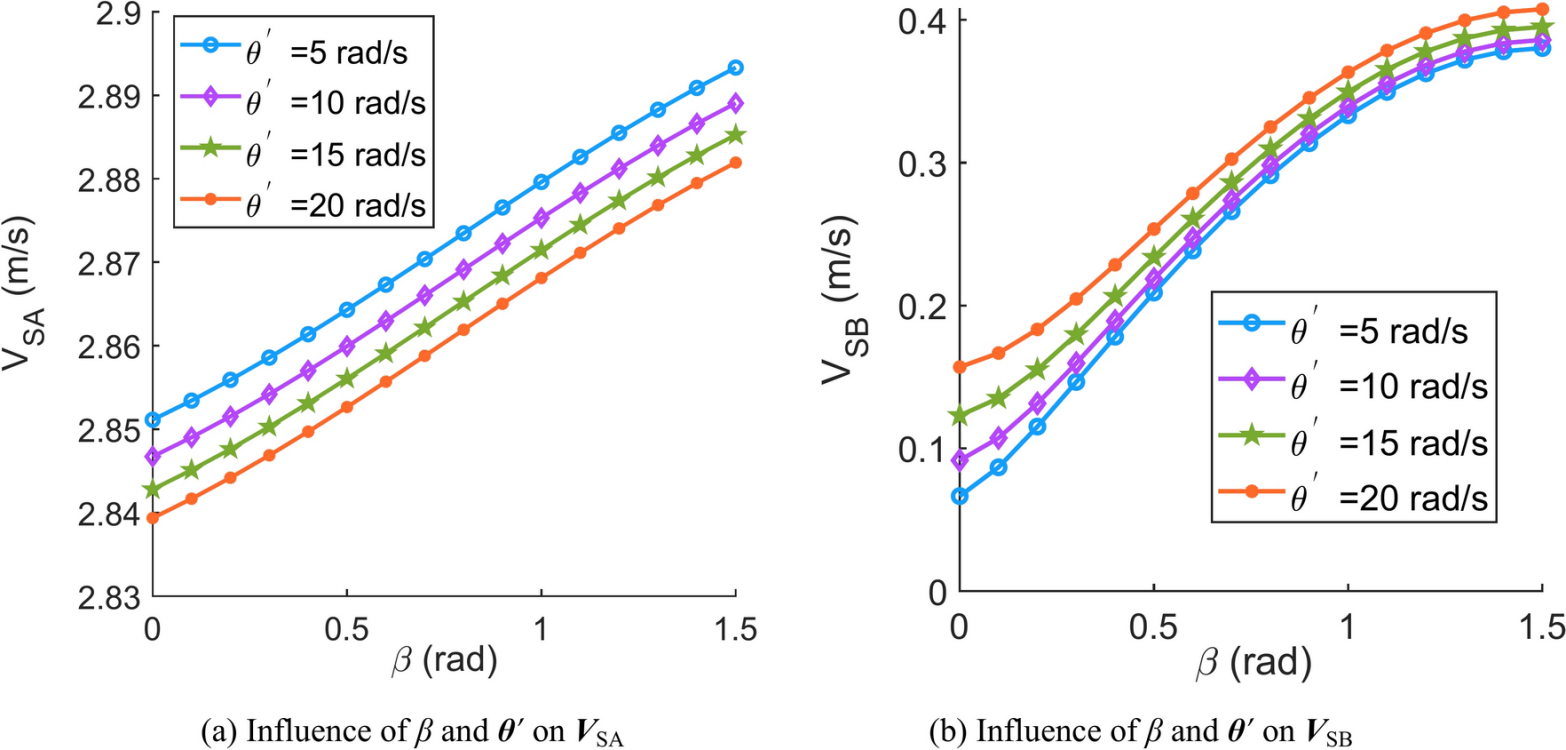
Influence of the contact angle (*β*) and angular velocity of the movable tooth relative to the central worm (***θ***^′^) on the sliding velocity of the movable tooth (***V***_S_).

**Fig. 9:**
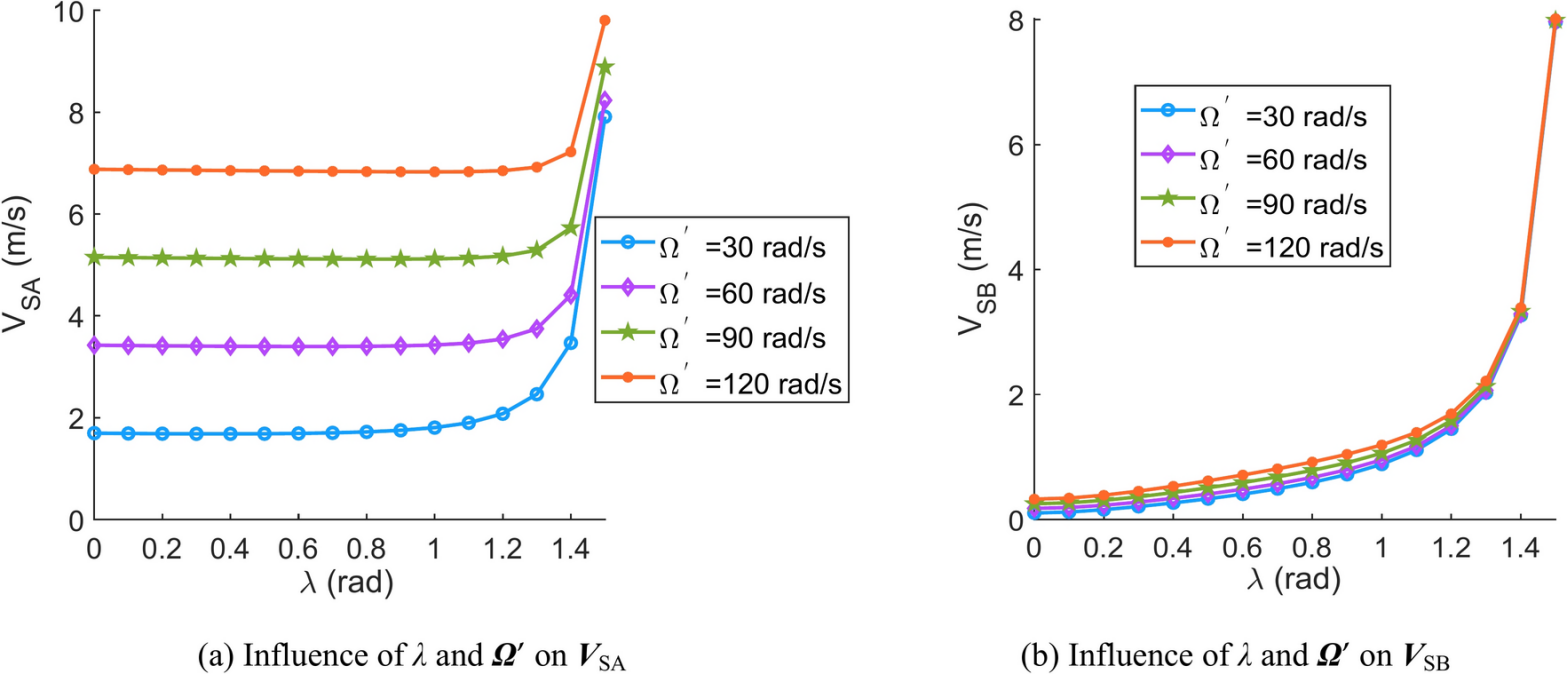
Influence of the spiral angle (*λ*) and rotation velocity of the central worm (***Ω***^′^) on the sliding velocity of the movable tooth (***V***_S_).

**Fig. 10:**
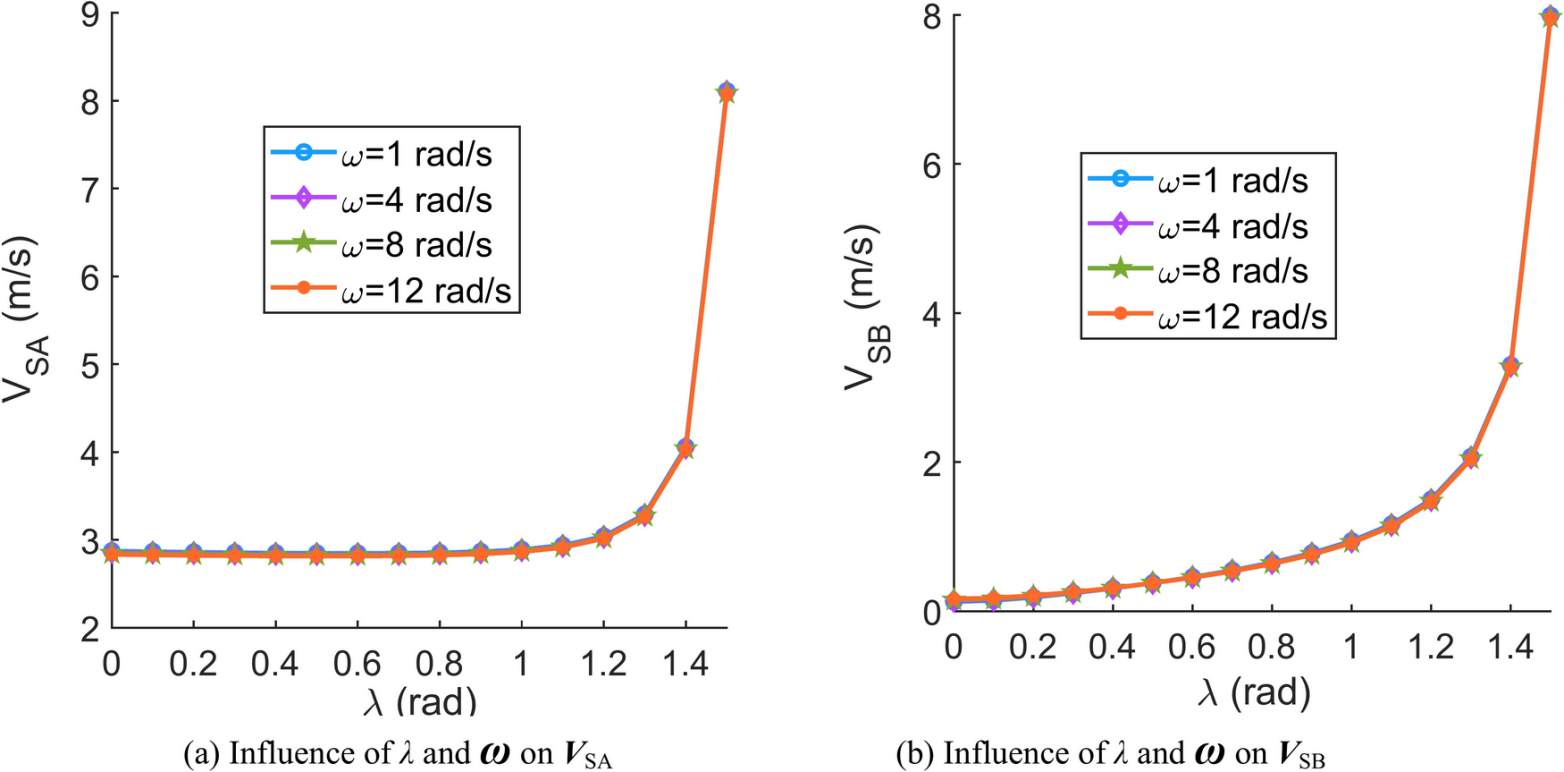
Influence of the spiral angle (*λ*) and spin velocity of the movable tooth (***ω***) on the sliding velocity of the movable tooth (***V***_S_).

**Fig. 11:**
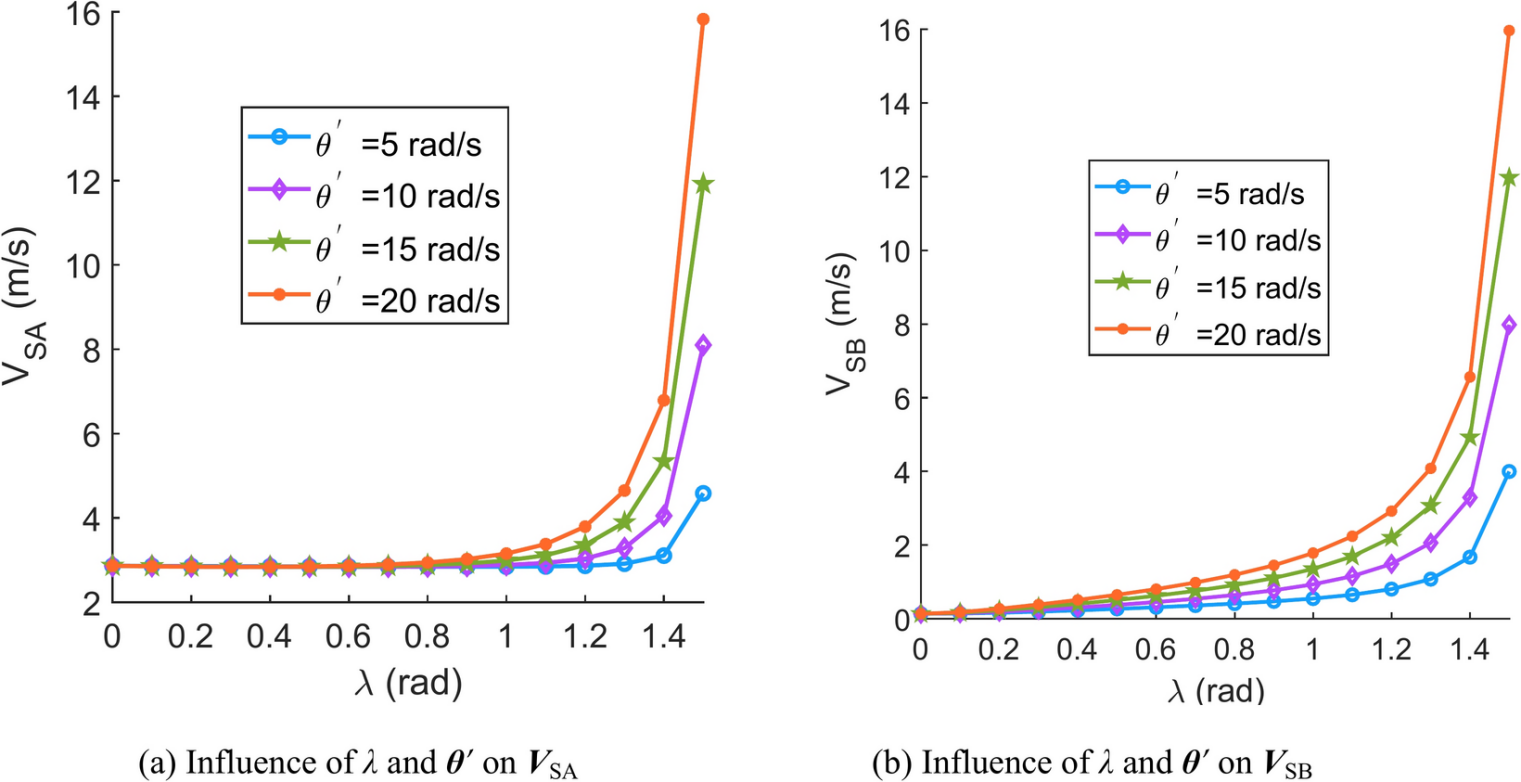
Influence of the spiral angle (*λ*) and angular velocity of the movable tooth relative to the central worm (***θ***^′^) on the sliding velocity of the movable tooth (***V***_S_).

According to Eqs. (31)–(32), the effects of important parameters (angular velocity of the central worm ***Ω***^′^, angular velocity of the movable tooth relative to the central worm ***θ***^′^, spin velocity of the movable tooth ***ω***, contact angle *β* and spiral angle of the central worm *λ*) on the sliding velocity are discussed in this section. When the parameters are set as Tables 1, 2, 3, 4, 5 and 6, the corresponding sliding velocity graphs of the movable tooth are shown in Fig. 6, 7, 8, 9, 10 and 11, respectively.

**Table 1:** Influence of the contact angle (*β*) and rotation velocity of the central worm (***Ω***^′^) on the sliding velocity of the movable tooth (***V***_S_).

Symbol	Description	Size	Units
*a*	Centre distance	119	mm
*Z*_2_	Teeth number of the planetary gear	8	–
*r*_2_	Radius of the circle in which the planetary gear teeth centre are located	62.5	mm
*ρ*	Radius of the movable tooth	8	mm
*λ*	Spiral angle of the central worm	*π*/24	rad
***ω***	Spin velocity of the movable tooth	4	rad/s
*θ*	Rotation angle of the movable tooth relative to the central worm	*π*/9	rad
*Ω*	Rotation angle of the central worm	*π*/6	rad
***θ***^′^	Angular velocity of the movable tooth relative to the central worm	10	rad/s

**Table 2:** Influence of the contact angle (*β*) and spin velocity of the movable tooth (***ω***) on the sliding velocity of the movable tooth (***V***_S_).

Symbol	Description	Size	Units
*a*	Centre distance	119	mm
*Z*_2_	Teeth number of the planetary gear	8	–
*r*_2_	Radius of the circle in which the planetary gear teeth are located	62.5	mm
*ρ*	Radius of the movable tooth	8	mm
*λ*	Spiral angle of the central worm	*π*/24	rad
***Ω***^′^	Rotation velocity of the central worm	50	rad/s
*θ*	Rotation angle of the movable tooth relative to the central worm	*π*/9	rad
*Ω*	Rotation angle of the central worm	*π*/6	rad
***θ***^′^	Angular velocity of the movable tooth relative to the central worm	10	rad/s

**Table 3:** Influence of contact angle (*β*) and angular velocity of the movable tooth relative to the central worm (***θ***^′^) on the sliding velocity of the movable tooth (***V***_S_).

Symbol	Description	Size	Units
*a*	Centre distance	119	mm
*Z*_2_	Teeth number of the planetary gear	8	–
*r*_2_	Radius of the circle in which the planetary gear teeth are located	62.5	mm
*ρ*	Radius of the movable tooth	8	mm
*λ*	Spiral angle of the central worm	*π*/24	rad
***ω***	Spin velocity of the movable tooth	4	rad/s
*θ*	Rotation angle of the movable tooth relative to the central worm	*π*/9	rad
*Ω*	Rotation angle of the central worm	*π*/6	rad
***Ω***^′^	Rotation velocity of the central worm	50	rad/s

**Table 4:** Influence of the spiral angle (*λ*) and rotation velocity of the central worm (***Ω***^′^) on the sliding velocity of the movable tooth (***V***_S_).

Symbol	Description	Size	Units
*a*	Centre distance	119	mm
*Z*_2_	Teeth number of the planetary gear	8	–
*r*_2_	Radius of the circle in which the planetary gear teeth are located	62.5	mm
*ρ*	Radius of the movable tooth	8	mm
*β*	Contact angle	*π*/10	rad
***ω***	Spin velocity of the movable tooth	4	rad/s
*θ*	Rotation angle of the movable tooth relative to the central worm	*π*/9	rad
*Ω*	Rotation angle of the central worm	*π*/6	rad
***θ***^′^	Angular velocity of the movable tooth relative to the central worm	10	rad/s

**Table 5:** Influence of the spiral angle (*λ*) and spin velocity of the movable tooth (***ω***) on the sliding velocity of the movable tooth (***V***_S_).

Symbol	Description	Size	Units
*a*	Centre distance	119	mm
*Z*_2_	Teeth number of the planetary gear	8	-
*r*_2_	Radius of the circle in which the planetary gear teeth are located	62.5	mm
*ρ*	Radius of the movable tooth	8	mm
*β*	Contact angle	*π*/10	rad
***Ω***^′^	Rotation velocity of the central worm	50	rad/s
*θ*	Rotation angle of the movable tooth relative to the central worm	*π*/9	rad
*Ω*	Rotation angle of the central worm	*π*/6	rad
***θ***^′^	Angular velocity of the movable tooth relative to the central worm	10	rad/s

**Table 6:** Influence of the spiral angle (*λ*) and angular velocity of the movable tooth relative to the central worm (***θ***^′^) on the sliding velocity of the movable tooth (***V***_S_).

Symbol	Description	Size	Units
*a*	Centre distance	119	mm
*Z*_2_	Teeth number of the planetary gear	8	–
*r*_2_	Radius of the circle in which the planetary gear teeth are located	62.5	mm
*ρ*	Radius of the movable tooth	8	mm
*β*	Contact angle	*π*/10	rad
***ω***	Spin velocity of the movable tooth	4	rad/s
*θ*	Rotation angle of the movable tooth relative to the central worm	*π*/9	rad
*Ω*	Rotation angle of the central worm	*π*/6	rad
***Ω***^′^	Rotation velocity of the central worm	50	rad/s

According to Figs. 6, 7, 8, 9, 10 and 11, it can be concluded that.

1. Within the design range of the contact angle (*β*), the sliding velocity of the movable tooth at the engagement point (***V***_S_) gradually increases as the contact angle increases.
2. The influence of the spiral angle (*λ*) on ***V***_SB_ is greater than that on ***V***_SA_; moreover, the spiral angle has little effect on ***V***_SA_ when *λ* < 1 rad.
3. Sliding velocities of the movable tooth at engagement points (***V***_SA_, ***V***_SB_) increase as the rotation velocity for the central worm (***Ω***^′^) increases, especially the sliding velocity of the moveable tooth at the contact point with the planetary gear (***V***_SA_), which is more strongly affected by ***Ω***^′^.
4. In equal terms, the sliding velocity of the movable tooth at the engagement point with the central worm (***V***_SB_) is smaller than that with the planetary gear (***V***_SA_). Consequently, the machining accuracy and lubrication condition of the planetary gear ball socket should be improved.

## Physical prototype

**Fig. 12:**
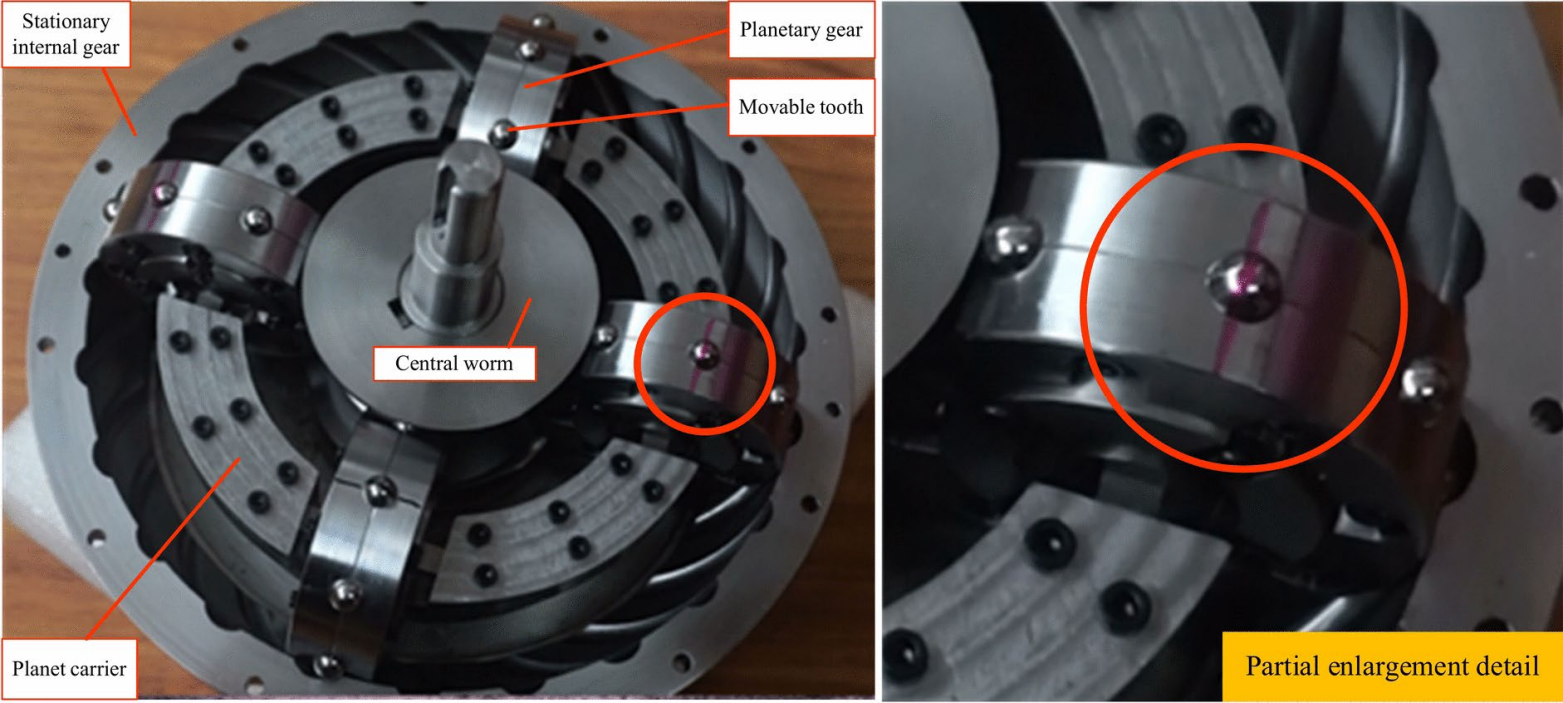
Initial state photo of the toroidal drive prototype.

**Fig. 13:**
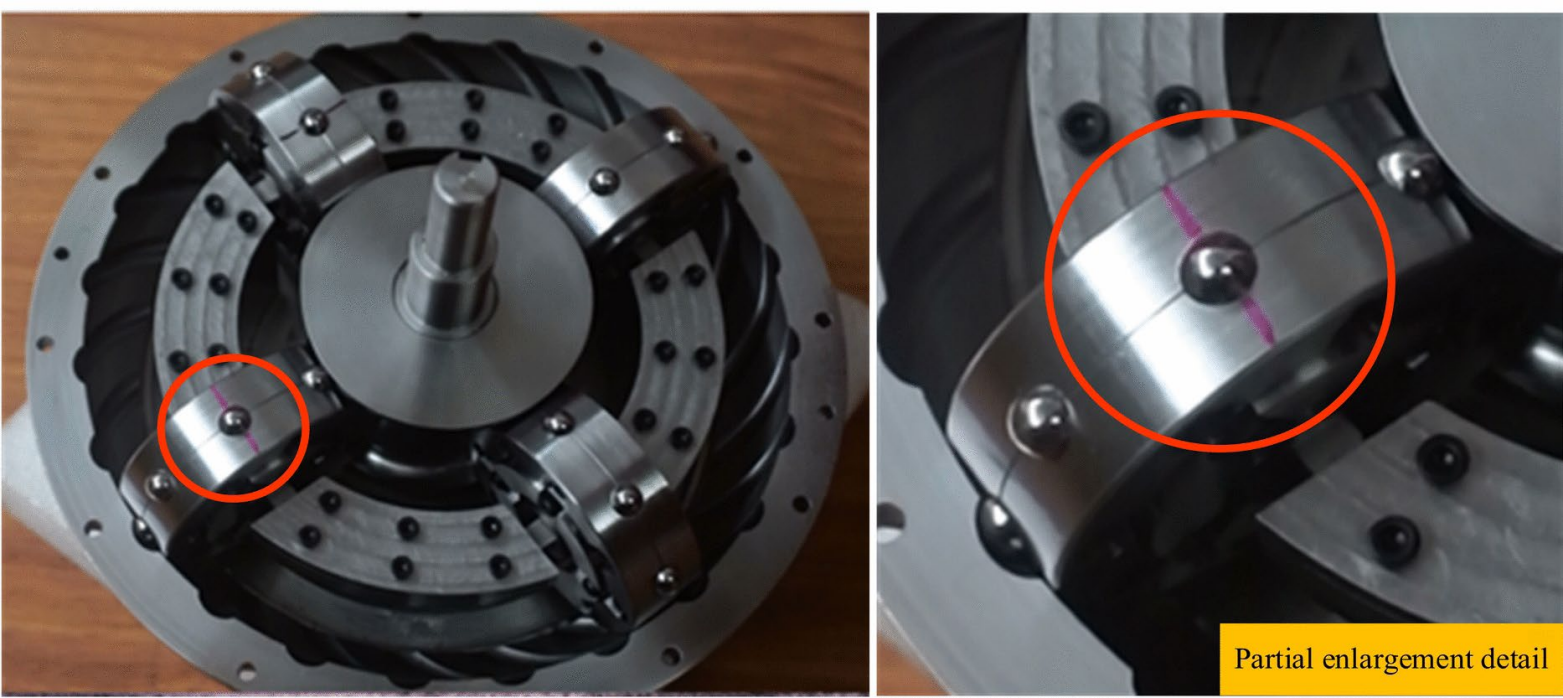
The state photo after the central worm rotates several turns.

A physical prototype of toroidal drive was produced to visually demonstrate the motion of spherical movable teeth, and the main design parameters are shown in Table 7. Figures 12 and Fig. 13 show the physical prototype of a toroidal drive with spherical movable teeth. Figure 12 shows the initial state of the toroidal drive when the red line on the movable tooth is collinear with that on the planetary gear. After the central worm rotates several turns, the red line on the movable tooth is no longer collinear with that on the planetary gear, which is due to the rolling and sliding of the movable tooth during the working process.

**Table 7:** Main design parameters of the physical prototype.

Design parameter	Numerical value
Centre distance (*a*)	59.5mm
Teeth number of the central worm (*Z*_1_)	1
Teeth number of the planetary gear (*Z*_2_)	8
Teeth number of the stationary internal gear (*Z*_3_)	23
Radius of the calculate circle for the central worm (*r*_1_)	26.7 mm
Radius of the calculate circle for the planetary gear (*r*_2_)	31.25 mm
Radius of the calculate circle for the stationary internal gear (*r*_3_)	92.3 mm
Radius of the movable tooth (*ρ*)	4mm

## Conclusions

In this work, the motion of a spherical movable tooth for a toroidal drive on a spiral raceway was studied. To describe the position of the movable tooth at the meshing point, the Frenet coordinate system and the concept of the contact angle were introduced into the toroidal drive. On the basis of the contact analysis of the spherical movable tooth, a rolling‒sliding mathematical model of the toroidal drive was established, thus providing a theoretical foundation for further friction loss analysis and transmission efficiency analysis of the toroidal drive.

The influence of the main parameters on the sliding velocity of the movable tooth was analysed. The results indicated the following. (1) Under the same conditions, the sliding velocity at the meshing point between the movable tooth and the planetary gear was greater than that between the movable tooth and the central worm. Therefore, more attention should be given to improving the surface finish and lubrication conditions of the planetary gear ball socket. (2) The sliding velocity increased with increasing central worm speed, especially at the meshing point between the spherical movable tooth and the central worm. (3) When the spiral angle of the central worm was less than 1 rad, the influence of the spiral angle on the sliding velocity at the engagement point of the planetary gear was weak.

## Data Availability

The data used to support the findings of this study is available from the corresponding author upon request.

## List of symbols

S_1_(*O*_1_*,x*_1_*,y*_1_*,z*_1_): World coordinate system of the movable tooth
S_1__′_(*O*_1__′_*,x*_1__′_*,y*_1__′_*,z*_1__′_): Rotating coordinate system of the movable tooth
S_0_(*O*,*t*,*n*,*b*): Frenet coordinate system of the movable tooth
***M***_1__′__1_: Coordinate transformation matrix from S_1_ to S_1__′_
*θ*: Rotation angle of the movable tooth relative to the central worm
*Ω*: Rotation angle of the central worm
*L*: Lead of the central worm
^*r*^***R***: Position vector of the movable tooth centre with respect to S_1__′_
*r*_*m*_: Instantaneous radius of the movable tooth trajectory
*λ*: Spiral angle of the central worm
^*w*^***R***: Position vector of the movable tooth centre with relative to S_1_
***R***(t): Trajectory curve of the movable tooth centre
*k*: Curvature of the trajectory curve for the movable tooth centre
*τ*: Torsion of the trajectory curve for the movable tooth centre
***M***_01__′_: Coordinate transformation matrix from S_1__′_ to S_0_
S_0_^′^: Variation rate of Frenet coordinate system
*β*: Contact angle
S_*i*_(*i*, X_*i*_, Y_*i*_, Z_*i*_): Additional coordinate system, *i* = A, B
***M***_*i*__0_: Coordinate transformation matrix from S_*i*_ to S_0_
***R***_AO_: Position vector of point A relative to the movable tooth centre
***R***_BO_: Position vector of point B relative to the movable tooth centre
^*r*^***R***_A_: Position vector of point A relative to the rotating coordinate system
^*r*^***R***_B_: Position vector of point B relative to the rotating coordinate system
***Ω***^′^: Angular velocity of the central worm
^*r*^***R***′: Velocity of the movable tooth with respect to the rotating coordinate system
^*w*^***R***′: Velocity of the movable tooth centre relative to the world coordinate
***ω***: Spin velocity of the movable tooth
***V***_Ar_: Instantaneous velocity of the movable tooth at contact point A
***V***_Br_: Instantaneous velocity of the movable tooth at contact point B
*φ*: Rotates angel of the planetary gear
*ρ*: Radius of the movable tooth
*a*: Centre distance
***V***_Aw_: Velocity of the planetary gear at contact point A
***V***_Bw_: Velocity of the central worm at contact point B
***V***_SA_: Sliding velocity of the movable tooth at contact point A
***V***_SB_: Sliding velocity of the movable tooth at contact point B
***θ***′: Angular velocity of the movable tooth relative to the central worm
*Z*_1_: Teeth number of the central worm
*Z*_2_: Teeth number of the planetary gear
*Z*_3_: Teeth number of the stationary internal gear
*r*_1_: Radius of the calculate circle of the central worm
*r*_2_: Radius of the calculate circle of the planetary gear
*r*_3_: Radius of the calculate circle of the stationary internal gear
